# Hopes and opportunities of stem cells from human exfoliated deciduous teeth (SHED) in cartilage tissue regeneration

**DOI:** 10.3389/fbioe.2023.1021024

**Published:** 2023-02-13

**Authors:** Forough Mahdavi-Jouibari, Benyamin Parseh, Ezatolah Kazeminejad, Ayyoob Khosravi

**Affiliations:** ^1^ Department of Medical Biotechnology, Faculty of Advanced Medical Technologies, Golestan University of Medical Sciences, Gorgan, Iran; ^2^ Stem Cell Research Center, Golestan University of Medical Sciences, Gorgan, Iran; ^3^ Faculty of Advanced Medical Technologies, Golestan University of Medical Sciences, Gorgan, Iran; ^4^ Dental Research Center, Golestan University of Medical Sciences, Gorgan, Iran; ^5^ Department of Molecular Medicine, Faculty of Advanced Medical Technologies, Golestan University of Medical Sciences, Gorgan, Iran

**Keywords:** mesenchymal stem cells, cartilage, regenerative medicine, tooth banking, SHED

## Abstract

Cartilage lesions are common conditions, affecting elderly and non-athletic populations. Despite recent advances, cartilage regeneration remains a major challenge today. The absence of an inflammatory response following damage and the inability of stem cells to penetrate into the healing site due to the absence of blood and lymph vessels are assumed to hinder joint repair. Stem cell-based regeneration and tissue engineering have opened new horizons for treatment. With advances in biological sciences, especially stem cell research, the function of various growth factors in the regulation of cell proliferation and differentiation has been established. Mesenchymal stem cells (MSCs) isolated from different tissues have been shown to increase into therapeutically relevant cell numbers and differentiate into mature chondrocytes. As MSCs can differentiate and become engrafted inside the host, they are considered suitable candidates for cartilage regeneration. Stem cells from human exfoliated deciduous teeth (SHED) provide a novel and non-invasive source of MSCs. Due to their simple isolation, chondrogenic differentiation potential, and minimal immunogenicity, they can be an interesting option for cartilage regeneration. Recent studies have reported that SHED-derived secretome contains biomolecules and compounds that efficiently promote regeneration in damaged tissues, including cartilage. Overall, this review highlighted the advances and challenges of cartilage regeneration using stem cell-based therapies by focusing on SHED.

## Introduction

Articular cartilage lesions, reported in nearly 36% of athletes and 63% of non-athletes, become more prevalent with advancing age and inadequate physical activity in young people (Fernandes et al., 2020). The lack of nerves and lymph makes the articular cartilage a particular tissue in the body (Sophia Fox et al., 2009; Simon and Jackson, 2018). Due to the absence of a vasculature which results in the reduced ability of cartilage tissue to repair themselves, these tissues cannot access growth factors required for cell regeneration (Zainal Ariffin et al., 2012). Despite recent advances, treatment of damaged cartilage seems unattainable (Frenkel and Di Cesare, 2004; Murphy et al., 2020). Owing to its inherent characteristics, cartilage has minimal access to humoral agents and potential restoration cells after injury (Hardingham et al., 2002; Frenkel and Di Cesare, 2004). Common treatments for damaged tissues include microfracture (Steadman et al., 2003), excision and drilling (Insall, 1967), chondrocyte, and osteochondral transplantation (autograft and allograft) (Pinski et al., 2016; Jackson et al., 2019). Although these procedures can alleviate the symptoms of patients, fibrocartilage tissues may develop which are mechanically distinct from a normal cartilage tissue (Simon and Jackson, 2018).

Cell-based therapies provide a novel approach in regenerative medicine to treat and repair damaged cartilage tissues. The use of mesenchymal stem cell (MSCs) and autologous chondrocyte implantation (ACI) is more common in cell therapy, and tissue regeneration approaches. ACI is a two-stage procedure involving arthroscopic removal of healthy cartilage, expansion of produced cells (cell culture-expanded), and the implantation of new cartilage (Niemeyer et al., 2014; Fernandes et al., 2018; To et al., 2019; Fernandes et al., 2020). However, differentiated cartilage cells have limited proliferative potential, and access to an adequate number of cells for transplantation is a major challenge. Additionally, the proliferative capacity of autologous cartilage cells decreases with aging, which may act as a significant barrier to the treatment of age-related cartilage disorders such as osteoarthritis (OA) (Dozin et al., 2002). Generally, MSCs are excellent candidates for regenerative medicine as they have advantages over other stem cells, and there are no ethical issues related to their production (Wei et al., 2013; Lv et al., 2014). MSCs have a fibroblast-like morphology and clonogenic capacity. Friedenstein first identified these cells in the rat bone marrow (BM) in 1966 (Friedenstein et al., 1966). Recently MSCs have been isolated from tissues including the BM (Shi and Gronthos, 2003; Soleimani et al., 2012), Adipose (Zuk et al., 2002), dental pulp (Gronthos et al., 2000; Pierdomenico et al., 2005), human exfoliated deciduous teeth (Miura et al., 2003), periodontal ligament (Seo et al., 2004), and umbilical cord (Kern et al., 2006).

**FIGURE 1:**
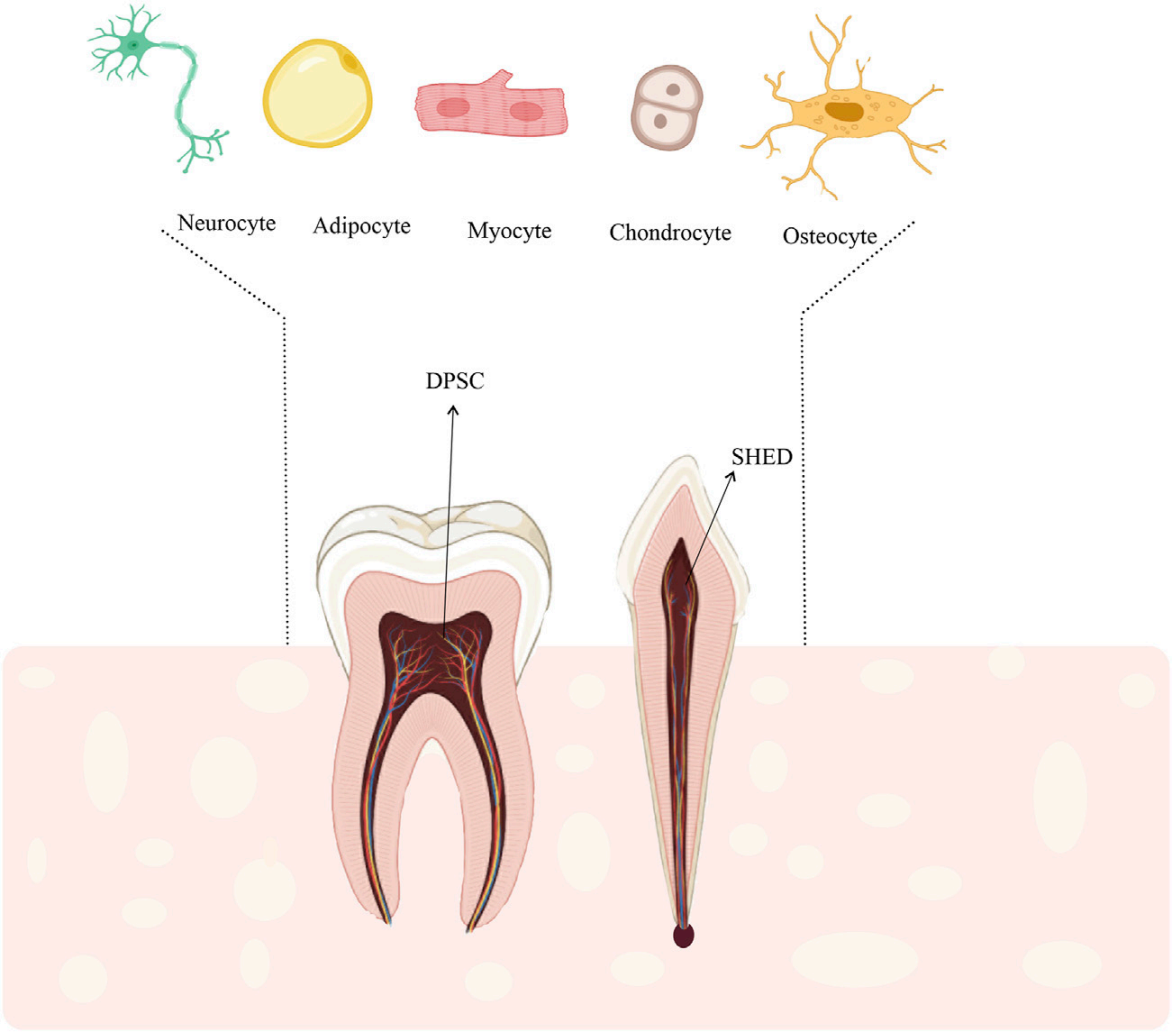
DPSCs and SHED could differentiate into other specified cells such as, adipocytes, myocytes, osteocytes, and chondrocytes. Therefore, SHED could be a suitable stem cell source for cell-based regenerative therapies including cartilage regeneration.

Microarray analysis showed that the dental pulp stem cells (DPSCs) and BM-MSCs express similar genes (Shi et al., 2001; Tamaki et al., 2013; Shen et al., 2019). One of the main differences between these two types of cellular sources is the high proliferation of DPSC compared to BM-MSC (Nakamura et al., 2009; Karaöz et al., 2011; Shen et al., 2019). On the other hand, repeated studies have shown that stem cells from human exfoliated deciduous teeth (SHED) have higher proliferation and survival potentials compared to other dental sources (Majumdar et al., 2016). Other advantages of SHED include painless cell collection and minimal risk of invasion (Kawashima, 2012; Fernandes et al., 2020). More importantly, SHED and DPSC have been shown to differentiate into mesodermal (Ishkitiev et al., 2010; Nourbakhsh et al., 2011; Gugliandolo and Mazzon, 2022; Kok et al., 2022), ectodermal, and endodermal lineage (Ishkitiev et al., 2010; Gugliandolo and Mazzon, 2022; Kok et al., 2022) (Figure 1). Despite the very high morphological similarity of deciduous cells to DPSCs, studies suggest that SHED has higher rates of differentiation and proliferation than DPSC (Nakamura et al., 2009; Wang et al., 2012). The proliferation rate of SHED is higher than that of DPSC, and the proliferation rate of DPSC is higher than that of BM-MSCs (SHED > DPSC > BM-MSC) (Huang et al., 2009). Regardless of their remarkable proliferative properties, these cells are used to reduce the risk of oncogenesis and modulate the immune system (Dai et al., 2019; Shen et al., 2019; Taguchi et al., 2019; Khosravi, 2020). Moreover, SHED-derived MSCs can be an available and potential source for tissue engineering and regenerative medicine (Wang et al., 2012; Taguchi et al., 2019). The present review aimed to introduce SHED as a viable option for cartilage regeneration.

### Cartilage lesions

While damage to articular cartilage is not life-threatening, it causes pain as it progresses and leads to a significant loss of movement, affecting the individual’s life (Semião, 2017). Depending on the underlying causes, two different phenotypes of cartilage damage have been categorized. Focal lesions are well-defined abnormalities that are commonly caused by trauma, osteonecrosis, or osteochondritis dissecans. Also, degenerative lesions are usually caused by osteoarthritis, meniscus injuries, deformities, or ligament instability (Falah et al., 2010). Morphological and biochemical changes in the tissue, caused by senescence, impair the mechanical properties and reduce the ability of chondrocytes to preserve the articular cartilage; besides, changes in the secretory phenotypes of cells can be detected (Loeser, 2009; Anderson and Loeser, 2010; Lotz and Loeser, 2012). The effects of these lesions on the synthesis and secretion of chemokines, cytokines, and proteases need to be characterized.

## Cartilage regeneration

### Tissue engineering

In the past, humans used to replace missing body parts with dead or artificial things of their own construction (Abdullah et al., 2013). Generally, the root cause of many diseases, neurological disorders, heart failure, and OA, is the absence of a significant cell population of cells that our bodies cannot replace (Murry and Keller, 2008). Although the application different surgical techniques have led to considerable progress, repair of damaged cartilage *via* proliferation or synthesis of natural hyaline has yet to be achieved (Niemeyer et al., 2014; Richter et al., 2016; Lee and Wang, 2017; Fernandes et al., 2018; Yang et al., 2018). Recent research has focused on the potential use of tissue and stem cell engineering in repair and reconstruction of bodily components (Nardi, 2009). It seems that by implementing the available tissue engineering knowledge and using suitable cells, tissue or organ reconstruction can be attained by scaffolding and morphogenic signals necessary for cell induction (Estrela et al., 2011; Zhang et al., 2019; Weizel et al., 2020). Stem cell-based tissue engineering has been recently advocated for cartilage repair (Pelttari et al., 2006; Craft et al., 2015; Guzzo and O’Sullivan, 2016; Nam et al., 2018).

### Stem cell therapy

It is well accepted that mature cartilage cannot be restored. The failure is caused by an insufficient inflammatory response following damage and the inability of stem cells to migrate to the injury site owing to the absence of arteries or lymph. However, in previous research when adult human chondrocytes were isolated from the native matrix and transplanted to human and animal models with cartilage defects or *ex vivo* culture conditions, they could produce cartilage-like extracellular matrix (ECM) (Ebihara et al., 2012; Van Pham, 2017). The discovery of stem cells has resulted in the development of novel therapeutic techniques for regenerating damaged tissues, as the distinguishing features of all stem cells after birth include multiplicity and self-renewal (Bianco et al., 2008). The direct injection of the cells into the affected area both prevents damage and causes tissue regeneration. Overall, the outcome of cell therapy is determined by the extent of the injury, injection method, mechanism of release, and dosage (Van Pham, 2017).

### Stem cell types for cartilage regeneration

Stem cells are derived from two primary sources: tissue-specific stem cells or adult stem cells (ASCs) (Bonab et al., 2006) and pluripotent embryonic stem cells (ESCs) (Thomson et al., 1998). With recent advances, researchers have been able to produce induced pluripotent stem cells (iPSCs) from embryonic and adult origin through reprogramming and defined protein and gene factors (Takahashi et al., 2007). Research on ESCs, iPSCs, and ASC, are at various stages of cartilage regeneration (Van Pham, 2017). The ESCs are derived from the intracellular body in the blastocyst stage. The iPSCs can be developed by reprogramming somatic cells. The ASC can be derived from different body tissues (Wang et al., 2017). Both ESCs and iPSCs can proliferate and develop into three germ layers, which are, ectoderm, mesoderm, and endoderm (Lee et al., 2013; Cheng et al., 2014). The ASC, can be classified into multipotent and unipotent types. Multipotent cells are divided into various types such as MSCs that can develop into chondrocytes, osteocytes, and adipocytes. On the other hand, unipotent stem cells can develop into only 1 cell lineage, such as satellite stem cells from skeletal muscle or epidermis (Dulak et al., 2015). Due to the absence of ethical restrictions and high availability, most clinical trials utilize MSC as a therapeutic agent for cartilage repair (Van Pham, 2017; Rathan et al., 2019). Overall, selection of the right source of MSCs depends on availability, easy preparation, and cartilaginous potential of each resource. Although there are many studies on the use of ESCs and iPSCs, there are still ethical and safety issues (i.e., immune stimulation, teratoma, and tumor) for the clinical application of these sources (Orth et al., 2014).

### Embryonic stem cells (ESCs)

In 1998, Thomson was the first to isolate human ESCs with significant telomerase expression. These cells can develop into all three germ layers, namely, ectoderm (i.e., neural epithelium, stratified squamous epithelium, and embryonic ganglia), mesoderm (i.e., bone, cartilage, smooth and striated muscle), and endoderm (i.e., gut epithelium) (Thomson et al., 1998). The identification of ESCs has revolutionized the field by introducing human embryogenesis and providing an unlimited potential source of therapeutic cells to treat various diseases (Van Pham, 2017; Sozen et al., 2018). According to a study by (Kramer et al. (2000), on mice, embryonic cell differentiation can be modulated *in vitro* into chondrocytes by transforming growth factor-β family (TGF-β1, BMP-2, and BMP-4). Additionally, (Nakayama et al. (2003) reported that ESCs produce different mesoderm cells when stimulated with BMP4. Among the derived cells, those expressing PDGFRα + or flk-1 showed cartilaginous activity in the presence of TGF-β3 and expressed cartilage-specific genes in seven-to 16-day culture. However, cells derived from early human embryos have raised significant ethical concerns as they inhibit embryonic development. Other challenges of this cellular resource include the immunization of ESCs in clinical applications (Van Pham, 2017).

### Induced pluripotent stem cells (iPSCs)

In 2006, Yamanaka et al. established iPSCs. Under ESC culture conditions, pluripotent stem cells from embryonic or adult mouse fibroblasts were generated using four transcription factors, including Oct3/4, Sox2, c-Myc, and Klf4. Homeobox protein Nanog has been confirmed to be unanticipatedly unneeded (Takahashi and Yamanaka, 2006; Nam et al., 2018; Karagiannis et al., 2019). In 2007, a comparable method for human fibroblasts was reported to generate human iPSCs by combining various components (Takahashi et al., 2007; Karagiannis et al., 2019). Generally, iPSCs have similar proliferation, replication, and gene expression characteristics to ESCs. However, unlike ESCs, these cellular resources are associated with no ethical limitations, as they are unique to each patient and can be easily produced from the individual’s somatic cells (Li et al., 2016; Nam et al., 2018). Although these cells are reproducible, the efficiency of the procedure is poor, with only nearly 1% of transfected fibroblasts transforming into iPSCs (Yamanaka, 2009). The iPSCs have been used in previous research to generate various therapeutic cells (Van Pham, 2017). Ko et al. (2014) conducted a study to determine the cartilaginous characteristics of human pluripotent stem cells and to evaluate the difference in cartilage formation between iPSCs and BM-derived MSCs. They used undifferentiated iPSCs to produce embryoid bodies (EBs). Following the dissociation of EBs into single cells, cartilage culture was performed in the presence of alginate hydrogel. Chondro-induced iPSCs were implanted in animals with osteochondral abnormalities and assessed 12 weeks. In chondrocytes generated by iPSCs, embryonic markers Nanog, SSEA4, and Oct3/4 disappeared, whereas BMP-4 emerged as a mesodermal marker. The main challenge of utilizing iPSCs for therapeutic and *in vitro* applications is achieving the required uniform cell differentiation and a single-sort cell lineage (Lietman, 2016). Therefore, to establish successful treatments for cartilage repair using iPSCs, a standard differentiation procedure with highly repeatable differentiation efficiency is required. Nevertheless, concerns about cancer in iPSCs, should be addressed in preclinical investigations using animal models (Van Pham, 2017).

### Mesenchymal stem cells (MSC)

The MSC_S_ can differentiate into specific cells within a lineage (Dua et al., 2003; Strauer and Kornowski, 2003). The benefits of these stem cells include low immune system activation, lack of tumorigenicity, and minimal ethical issues (Seyhoun et al., 2019). However, limited differentiation is a significant barrier against the therapeutic application of MSCs (Zuk et al., 2002; Szychlinska et al., 2017). It is known that MSCs migrate through cerebrospinal fluid toward damaged spinal cord tissue and integrate with the host tissue following migration. These cells are expected to play a promising role in tissue regeneration for cell-based therapy (Satake et al., 2004; Woo et al., 2020; Zhang et al., 2020). The International Society for Cell Therapy (ISCT) has recommended three criteria for classifying MSCs, which are as follows (Fernandes et al., 2020): MSCs must be adherent under conventional culture conditions (Simon and Jackson, 2018), they express surface antigens such as CD105, CD90, CD73, and they do not express CD45, CD34, CD14 (CD11b), CD19 (CD79a), and HLA-DR (Sophia Fox et al., 2009). The MSCs can differentiate into osteogenic, chondrogenic, and adipogenic cell lineage according to these criteria (Dominici et al., 2006). It is well established that MSCs can be found in almost all post-natal tissues and may be obtained from various tissues such as skeletal muscle (Williams et al., 1999), BM (Haynesworth et al., 1992), umbilical cord (Erices et al., 2000), placenta (In’t Anker et al., 2004), and adipocyte (Halvorsen et al., 2000; Go et al., 2020). The first isolated human dental MSCs were derived from pulp tissue (Gronthos et al., 2000). Followed by other dental MSCs such as SHED (Miura et al., 2003), periodontal ligament stem cells (PDLSCs) (Seo et al., 2004), and dental follicle progenitor/stem cells (DFPSCs) (Morsczeck et al., 2005) with ectomesenchymal origin have been identified. These cells have different differentiation potentials, replication rates, and surface marker properties (Morsczeck et al., 2005; Strioga et al., 2012; Abu Kasim et al., 2015; Anitua et al., 2018; Yamada et al., 2019). Dental stem cells express specific markers expressed by ESCs and MSCs, such as oct4, CD106, STRO-1, and NANOG (Gronthos et al., 2000; Ferro et al., 2012). Under certain stimuli, these clonogenic cells can differentiate into different cells, such as neurons, adipocytes, odontoblasts (Miura et al., 2003; Pivoriūnas et al., 2010; Wang et al., 2010), osteocytes, and chondrocytes (Dai et al., 2012).

## Tissue reservoirs of MSCs

### Bone marrow

The MSCs originate from the BM and differentiate into various mesodermal cell types, including bone, cartilage, adipose, and muscles (Prockop, 1997). The MSCs from the BM tissue can be used to repair the cartilage, making this tissue one of the most common sources of MSCs. It should be noted that BM-MSCs are extremely rare; consequently, they are not the richest source of stem cells (Bonab et al., 2006). Besides, the number of BM-MSCs decreases with advancing age, and cell therapy becomes more difficult. Aspiration, on the other hand, involves an invasive and painful process of cell removal from the BM and increases the risk of infection (Dao et al., 2017). According to investigations by McCarthy et al. (2012), BM-MSCs are prone to the formation of osteophytes, which are subchondral bone overgrowths. They may also develop hypertrophic cartilage phenotypes and eventually differentiate into calcified cartilage; accordingly, research into other MSC types is ongoing.

### Adipose tissue

Zuk et al. (2001) discovered ADSCs by liposuction in 2001. ADSCs can be distinguished from BM-MSCs by their differentiation capacity, cell surface markers, and abundance in the body. Since more stem cells can be derived from the adipose tissue than BM, ADSCs have a significant functional advantage over BM-MSCs (Aust et al., 2004; Oedayrajsingh-Varma et al., 2006). The isolation of MSCs from the adipose tissue consists of a number of simple processes that can be performed by anyone with cell culture laboratory experience (Estes et al., 2010). The limitations of the adipose tissue are its lower capacity for cartilage development compared to BM and the presence of embryonic markers, such as Oct-4, Nodal, and Utf-1 (Jonidi Shariatzadeh et al., 2018).

### Wharton’s jelly

The MSCs derived from Wharton’s jelly have significant applications in regenerative medicine. These MSCs express markers of both EMSCs and MSCs and may be collected painlessly and safely from the donor site. These cells have other benefits, including rapid proliferation, limitless multipotency, minimal immunogenicity, and most importantly, lack of tumorigenicity (Fong et al., 2010; Gauthaman et al., 2012). The Wharton’s jelly has been successfully differentiated into the chondrogenic lineage in different studies using chondrogenic factors (Wang et al., 2011; Reppel et al., 2015); however, this source may have high initial banking costs (Moise, 2005).

### Stem cells from human exfoliated deciduous teeth (SHED)

Multiple populations of MSCs have been identified and isolated from dental and oral tissues, including PDLSCs, DPSCs, papillary apical stem cells (SCAP), DFPSCs, gum-derived stem cells (GMSCs), and SHED. Due to their easy accessibility, DPSCs and SHED have received the greatest attention (Yang et al., 2019; Shi et al., 2020). In this regard, a study of oral stem cells and BM-MSCs reported that SHED has the highest reproductive potential, followed by GMSCs, while BM-MSCs had the lowest reproductive potential (Li et al., 2018). Masako Miura et al. isolated SHED in 2003. Generally, SHED can be easily obtained from readily available dental pulp tissue with no major ethical concerns and provide adequate cells for clinical applications due to strong proliferation and telomerase expression (Miura et al., 2003). Similar to DPSCs, SHED expresses different markers, including CD13, CD44, CD73, CD90, CD146, CD166, and STRO-1 (Miura et al., 2003; Govindasamy et al., 2010; Akpinar et al., 2014), but not CD34, CD45, or HLA-DR (Karaöz et al., 2010; Akpinar et al., 2014). Another hallmark that indicates the mesenchymal origin of these cells is the lack of markers associated with the monocytic and hematopoietic lineage. The SHED and DPSCs, similar to ESCs, have been shown to have pluripotency markers, including Oct-4, Nanog, Sox-2, as well as insulin-like growth factor-1 receptor (IGF1R) (Karaöz et al., 2010; Kaukua et al., 2015). These transcription factors are essential for cell proliferation and self-renewal. Studies suggest that Oct-4 and Nanog regulate MSC proliferation and differentiation (Tsai and Hung, 2012). Although SHED has some similarities to DPSCs, they have different gene expression pattern (Galler et al., 2008; Nakamura et al., 2009). SHED has higher levels of pluripotent markers, such as Oct-4, Nanog, and Sox-2 compared to DPSC (Govindasamy et al., 2010). Compared to DPSC and even BM-MSCs, SHED also has a higher proliferation rate (Miura et al., 2003; Nakamura et al., 2009). Moreover, the mineralization ability of SHED is higher than that of DPSC. The mRNA expression levels of inflammatory cytokines, such as interleukin-6 (IL-6), and other proteins such as matrix metalloproteinase 1 (MMP1), tissue inhibitor of metalloproteinase 1 (TIMP1), MMP2, and TIMP2, were significantly higher than in DPSC_S_ (Wang et al., 2012).

### Immune-modulatory properties of SHED

Recently, major attention has been paid to the immunomodulatory characteristics of dental MSCs. According to previous research, the application of allogeneic cells may result in host immune system rejection due to tissue incompatibility. Nevertheless, MSCs can modulate the immune system by secreting soluble factors, enzyme expression, and cell-to-cell interaction (Pierdomenico et al., 2005; Li et al., 2014; Woo et al., 2020). Yamaza et al. (2010) discovered that SHED had a significant effect on the suppression of the T-helper 17 cells *in vitro* compared to the immunomodulatory properties of BM-MSCs. Moreover, de Sá Silva et al. (2014) found that when monocyte-derived dendritic Cells (moDCs) were cultivated on SHED, the production of pro-inflammatory cytokines namely IL-2, interferon-λ (IFN-λ), and tumor necrosis factor-α (TNF-α) decreased while the expression of IL-10 as anti-inflammatory cytokine, increased. Additionally, Yu-Yang Dai et al. performed *in vivo* and *in vitro* investigations on the immunomodulatory properties of SHED for the treatment of allergic rhinitis (AR). In the SHED administration group, the Th2-mediated responses, nasal symptoms, and inflammatory responses significantly decreased. Besides, peripheral blood mononuclear cells (PBMCs) from AR patients were cultivated with SHED and BM-MSCs in the presence of phytohemagglutinin. It was found that SHED inhibited T-cell proliferation, decreased the production of mediators including IL-4 and IL-17A, and increased the Th1/Th2 ratio by stimulating Treg cell expansion (Dai et al., 2019). Numerous studies have confirmed the immunomodulatory effects of SHED *in vivo*. In this regard, (Gao et al. (2018) demonstrated that the injection of SHED in the mouse periodontitis model caused bone regeneration and led to the suppression of the inflammatory response, as well as immune response modulation. They proposed that the therapeutic effects of SHED were caused by the polarization of M2 macrophages. According to previous findings, SHED modulates the immune system by regulating immune cell proliferation and differentiation and adjusting the expression of pro-inflammatory and anti-inflammatory mediators.

### Tumorigenicity risk of SHED

In most settings, genomic changes are an inevitable phase of *in vitro* stem cell culture. The frequency of these changes, which increases over time in the culture, represents at least one general process of increased tumorigenicity (Maitra et al., 2005), which is a particularly critical gap in the application of these cells. Overall, stem cell tumorigenicity is the primary barrier to the safe application of stem cell-based regenerative medicine. Although some of adult stem cell therapies may appear safe, they have limited applications for the treatment of human disease (Knoepfler, 2009). According to a recent study in 2020 by Yuk Wah Chan et al., despite the tremendous therapeutic potentials of adipose-derived stem cells (ADSCs), they spontaneously fused with breast cancer cells (BCC); these hybrids exhibit surface antigen markers of cancer stem cells (CSCs) and showed strong *in vivo* tumorigenic capacity. CSCs are a rare population in tumors with high metastatic potential and resistance to treatment (Khosravi et al., 2021). Recent studies have shown the ability of MSCs to regulate CSCs through increased tumor-initiating abilities, enhance the resistance of CSCs to chemotherapy and drive the metastatic *preferences* of CSCs to specific tissues (Cuiffo and Karnoub, 2012). Therefore, the predilection of MSCs to cooperate with CSCs in tumor initiation, progression and metastasis should be further investigated. Several studies have reported that ADSCs or MSC increase the BCC migration and proliferation either through mediators and growth factor secretion or directly *via* cell fusion (Chan et al., 2020). Wen-Ching Shen et al., in a study on tumorigenesis in BM cells and DPSCs, found that a higher PTEN expression in DP-MSCs relative to BM-MSCs was responsible for differences in the lineage linkage and tumorigenesis of these two cell types. Moreover, the PTEN promoter in BM-MSCs exhibited higher DNA methylation levels compared to DP-MSCs, besides enrichment of histone H3 lysine 9 dimethylation (H3K9Me2,) mediated by enhanced Dnmt3b and G9a expression. The results showed how multiple epigenetic variables influence the lineage linkage and tumorigenesis in various ways. According to the findings, these variables need to be considered in the development of treatments based on stem cells (Shen et al., 2019).

### SHED-based strategies for cartilage regeneration

**FIGURE 2:**
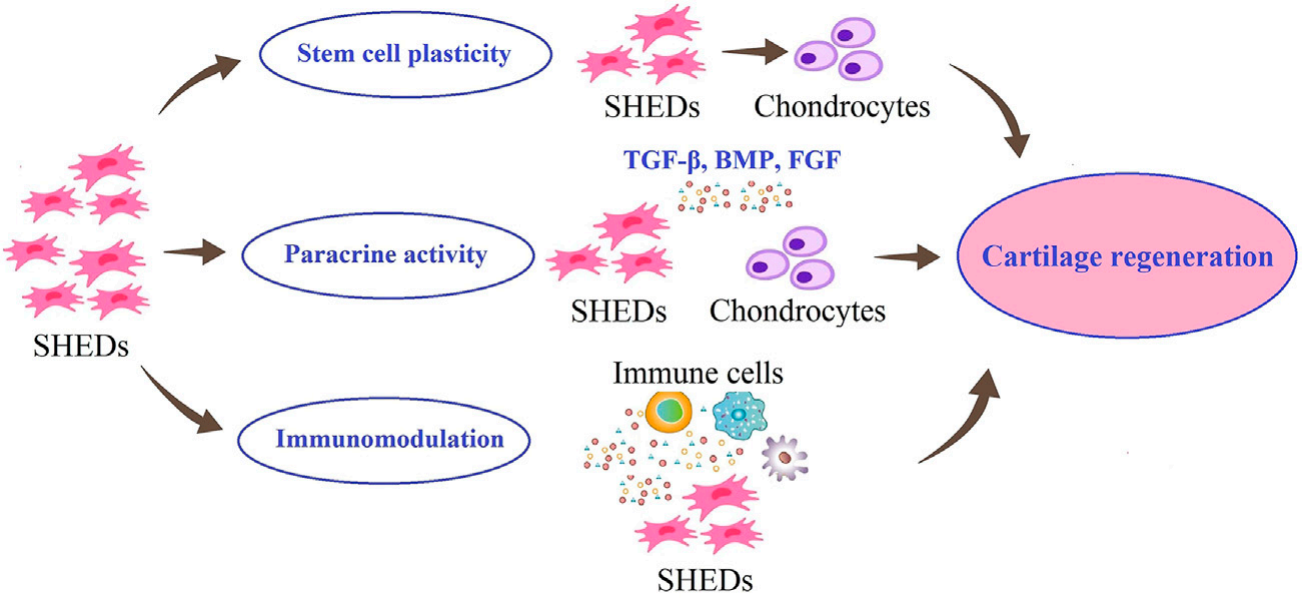
Possible roles of SHED in cartilage regeneration. SHED can proliferate and differentiate into chondrocytes. Also, SHED can secrete different growth factors, to maintain chondrocyte phenotypes and promote their proliferation. In addition, SHED have immunomodulatory effects on the lesion site.

The selection and development of SHED-based strategies for regeneration of damaged cartilage tissues require understanding the molecular mechanisms involved in cartilage regeneration. These strategies are shown schematically in Figure 2.

The first strategy can be the use of endogenous MSCs of damaged cartilage. Previous studies have shown that the quantity and potency of endogenous MSCs are insufficient to regenerate cartilage repair completely (Baraniak and McDevitt, 2010). Considering the possibility of migration of transplanted MSCs to damaged areas and differentiation into target cells (Pan et al., 2020), researchers are looking for exogenous MSCs with promising potential for migration, proliferation, and differentiation into chondrocytes. SHED have been reported to have the chondrogenic differentiation ability in both *in vitro* and *in vivo* models, suggesting their potential of them in cartilage regeneration (Chen et al., 2014).

The second strategy in cartilage regeneration is to use the therapeutic effects of SHED through their paracrine effects. Extracellular vesicles (EVs), exosomes and soluble growth factors, known as the secretome or serum-free conditioned media of SHED have been shown in numerous studies to have a modulatory effect on other cells or tissues, demonstrating the efficacy of cell-free based stem cell therapy in cartilage regeneration and maintain chondrocyte phenotypes and promote their proliferation (Guo and Yu, 2022). SHED can promote the proliferation of chondrocytes and the production of cartilage matrix by releasing nutritional factors, such as transforming growth factor beta (TGF-β), bone morphogenetic proteins (BMPs), and fibroblast growth factors (FGFs) (Bar et al., 2021).

A damaged cartilage is exposed to a progressive inflammatory environment. Evidence suggests that remodeling and reconstruction in the cartilage lesions that results from cell transplantation depends not only on differentiation potential but also on anti-inflammatory paracrine mechanism that reduce inflammation at the lesion site (Guo and Yu, 2022). The third strategy in cartilage regeneration could involve the role of SHED in modulating the immune response in the cartilage repair process. Yamaza et al. (2010) showed that the optimal therapeutic effect of SHED may be due to their immunomodulatory effects of them in terms of recovering the Tregs/Th17 ratio and reducing Th17 cell levels in peripheral blood. Studies have also shown that SHED have low expression of major histocompatibility complex class I (MHC I) and negative expression of major histocompatibility complex class II (MHC II) (Dai et al., 2019). These properties could support the use of SHED for immune modulation in clinical practice.

### Efficacy of SHED in cartilage regeneration

Regenerative medicine for functional tissue restoration has evolved from *ex vivo* tissue engineering to induction and intra-tissue restoration. Multidisciplinary approaches to cell biology and evolution, bioengineering, immunology, and genomic and proteomic elements of molecular biology have led to significant advances in regenerative medicine and tissue engineering (Naranjo et al., 2016). Also, biotechnology and regenerative medicine advances, along with the application of stem cells for tissue regeneration have resulted in significant medical progress (Sylvester and Longaker, 2004). The optimal cellular source for tissue engineering should be available and easily expanded *in vitro*. For instance, cartilage regeneration must produce significant extracellular matrix components without becoming dedifferentiated; chondrocytes and stem cells have been reported to be the primary resource for this purpose (Sharma and Elisseeff, 2004; Kock et al., 2012). Lars Patterson from the University of Gothenburg published research in 1987 which revealed that chondrocytes might be stimulated to proliferate *in vitro* and then utilized to repair cartilage lesions in the joint from which they have been originated. This method was developed by Genzyme Laboratories in Cambridge, UK, where arthroscopically obtained cells are still sent for culture (Manfredini et al., 2007). The only method approved by the Food and Drug Administration (FDA) for cartilage tissue engineering is the Carticel transplantation of *in vitro* expanded autologous chondrocytes (Wood et al., 2006; Camarero-Espinosa et al., 2016). This strategy utilizes the cartilage cells, conducts *in vitro* expansion, and implants the cells in the operated knee (Manfredini et al., 2007). Because differentiated cells cannot reflect all phenotypic changes in the tissue, the application of chondrocytes for tissue engineering is not ideal. Besides, the application and implantation of a clonal or immortal cell lineage can result in tumor development due to the unlimited proliferation of these cells (Camarero-Espinosa et al., 2016).

Some adverse effects of Carticel methods are graft failure, delamination, and tissue hypertrophy (Wood et al., 2006). Basic scientific studies and clinical trials have been conducted to apply stem cells and growth factors to restore damaged tissues (Strauer and Kornowski, 2003). Stem cells with paracrine activity and the release of vesicles and exosomes play a vital role in tissue regeneration and repair (Ando et al., 2014; Kay et al., 2017; Bjørge et al., 2018; Niazi et al., 2020). Dental epithelial cells and MSCs are great resources employed in dentistry and various regenerative medical disciplines. However, if these biological resources are not employed properly, they should be discarded (Xiao and Nasu, 2014; Mattei et al., 2021). The SHED can differentiate into bone, generate teeth, and develop into various non-dental mesenchymal cell lineages *in vitro* (Abdullah et al., 2013; Mattei et al., 2021). Ishikawa et al. demonstrated that injecting SHED-conditioned medium (SHED-CM) into mice could decrease symptoms of cartilage disease *via* the paracrine mechanism. It has been shown that SHED-CM suppresses inflammation and bone degradation. Therefore, it may provide an anti-inflammatory and restorative therapy for individuals with rheumatoid arthritis (Ishikawa et al., 2016). In a study by Werle et al. (2016), comparing the rate of proliferation and differentiation between healthy deciduous teeth and decayed deciduous teeth, the two sources of SHED had similar proliferation rates. Both cells expressed CD90, CD73, and CD29 markers, whereas the CD45, CD34, CD14, and HLA-DR were negative; they could differentiate into bone, cartilage, and adipose tissue cell lineages. Additionally, Chen et al. (2014) evaluated cartilage differentiation potential of SHED. In this study, SHED was cultured in a cartilage differentiation medium containing TGF-b3, basic fibroblast growth factor (bFGF), dexamethasone, insulin, and ascorbate phosphate for 2 weeks. The expanded SHED grown on the β-tricalcium phosphate (β-TCP) scaffold was transplanted into the subcutaneous space on the back of nude mice. Chondrogenesis was studied using Safranin O staining and toluidine blue staining. They also evaluated type II collagen and aggrecan *via* immunohistochemistry. The results revealed the *in vitro* and *in vivo* differentiation capacity of SHED into cartilage.

### Signaling molecules involved in chondrogenesis during cartilage development

MSCs originate from the mesoderm and form the appendicular skeleton (Camarero-Espinosa et al., 2016). Skeletal development is divided into four stages, the first of which is the migration of cells to the eventual skeletal site. Tissue interactions (epithelial-mesenchymal) occur in the following step. Cell condensation and differentiation into osteoblasts or chondroblasts are the final steps in the process. The cell condensation stage is critical for bone morphogenesis and mesenchymal tissues (Hall and Miyake, 2000; Chan et al., 2021). MSCs increase mitosis during organ development due to cell-cell or cell-substrate interactions, resulting in cell condensation (Camarero-Espinosa et al., 2016). The cartilaginous anlage is formed when stem cells in the condensation develop into chondrocytes, which produce an abundance of ECM proteins such as collagen types II, IX, and XI and proteoglycans (Demoor et al., 2014). During the endochondral ossification process, chondrocytes in the condensation zone become hypertrophic and express markers of terminal differentiation markers including type X collagen, Runt-related transcription factor 2 (RUNX-2), Indian hedgehog (Ihh) and MMP-13 (Mariani et al., 2014; Kwon et al., 2016). Several complicated regulatory mechanisms such as BMP, FGF, TGF-β, and Wnt signaling pathways are involved in chondrocyte destiny, determining whether they retain cartilage form or undergo hypertrophic maturation prior to ossification (Minina et al., 2002). Several TGF-β isoforms are activated during the cartilage regeneration process (van der Kraan and van den Berg, 2007). Proteoglycan and type II collagen are produced at a higher rate as a result of this activation, which also prevents ECM degradation (Darling and Athanasiou, 2005; Pogue and Lyons, 2006). TGF-β promotes cell proliferation and upregulated cartilage-specific genes such as glycosaminoglycans (GAG), aggrecan, and type II collagen. It has been demonstrated that SRY-related high-mobility-group box transcription factor (SOX) 9 mediated transcription is stimulated by TGF-β-activated Smad3/4 (Furumatsu et al., 2009; Kwon et al., 2016). TGF-β operates as a stimulator during the first stages of chondrogenesis but as an inhibitor throughout the suppressing the production of osteocalcin, MMP-13, type X collagen, and vascular endothelial growth factor (VEGF) in advanced phases of chondrocyte differentiation and prevents differentiate into hypertrophic chondrocytes (Mackie et al., 2008; Mariani et al., 2014).

**FIGURE 3:**
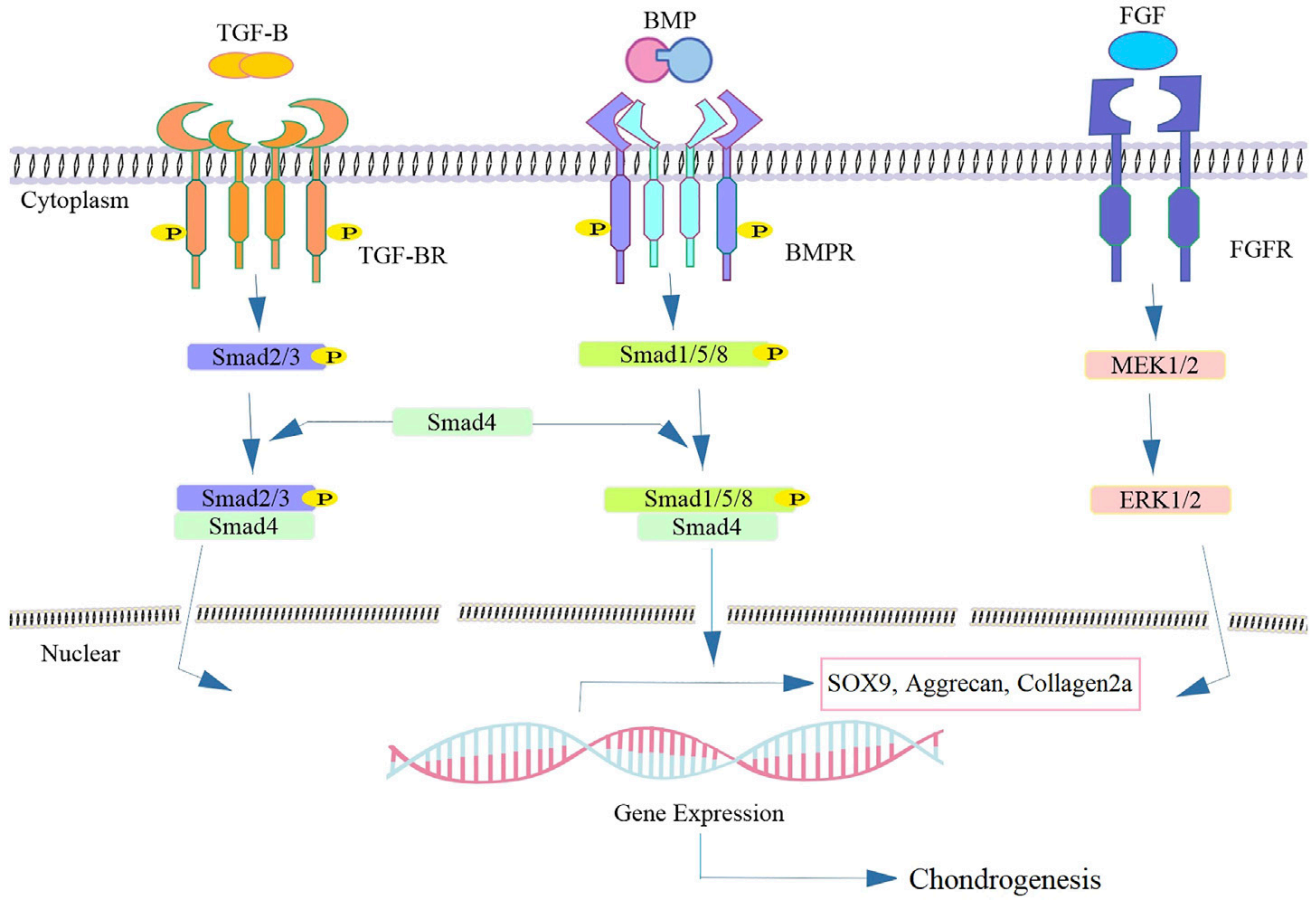
Schematics of representative signaling biomolecules present in SHED-CM responsible for cartilage regeneration. Following the binding of the TGF-β or BMP ligand to TGF-β-RII, leads to the phosphorylation of TGF-β-RI and subsequent phosphorylation and activation of the intracellular Smad superfamily. The heterocomplex of Smad4 and other co-activators translocate to the nucleus and thereby regulating the target gene expression such as sox9 involved in cartilage regeneration. FGF binds to FGFR and activates the downstream cascade and leading to the expression of the chondrogenic markers *via* activation of the MEK/ERK signaling pathway.

In embryonic skeletal development, BMPs are growth factors required for cartilage formation (Tsumaki et al., 2002). BMPs control the growth and resorption of cartilage as a member of the TGF family. BMPs signaling pathway is transduced Smad1/5/8 whereas Smad2/3 are responsible for mediating TGF-β signaling (De Caestecker, 2004) (Figure 3). Prechondrogenic condensation and chondrocyte maturation begin with the activation of BMP signaling (Mihelic et al., 2004). BMP-2 has been confirmed to have a substantial anabolic impact on cartilage. It has been shown that the upregulation of type II collagen by chondrocytes seeded in alginate, stimulates proteoglycan production in murine cartilage (Gründer et al., 2004). Regarding the protective impact of BMPs in cartilage regeneration; it has been demonstrated BMPs are related to matrix degradation and chondrocyte hypertrophy (Leboy et al., 2001). However, enhanced matrix turnover may be useful in replenishing matrix molecules during cartilage matrix regeneration. Enhanced matrix turnover may be useful in replenishing matrix molecules during cartilage matrix regeneration (Blaney Davidson et al., 2007). BMP-2 regulates the expression and function of SOX9 to enhance chondrocyte proliferation and matrix production, and regulate chondrogenesis (Zehentner et al., 1999). FGF2, also known as bFGF, is another factor involved in the healing of cartilage lesions. It is present in the pericellular matrix of cartilage at relatively high concentrations (Chia et al., 2009). FGFs enhance the amount of SOX9 expression and upregulate the type II collagen gene expression through increase the activity of SOX9-dependent, chondrocyte-specific enhancer elements (Figure 3). It binds to cell receptors and promotes anabolic signaling pathways, resulting in reduced aggrecanase activity, without any significant change in the proteoglycan composition (Fortier et al., 2011).

### SHED-CM promote cartilage regeneration

Cell-free therapies have been recently employed in tissue engineering and regenerative medicine to eliminate any concerns related to the use of stem cells (Vizoso et al., 2017; Harrell et al., 2019; Kumar et al., 2019; González-González et al., 2020; Parseh et al., 2022). Activated chondrocytes in the damaged site produce more cartilage, proinflammatory mediators, catabolic enzymes, and oxidative stress stimulators. This causes the cartilage matrix to degrade and induce chondrocyte apoptosis or necrosis by activating the NF-kB pathway, which results in severe articular injury and functional disability (Lo Monaco et al., 2020; Palamà et al., 2020). NF-kB transcription factors regulate a variety of immunological responses, survival, cellular differentiation, and growth in both healthy and malignant situations. Activation of NF-kB in chondrocytes controls the production of several matrix-degrading enzymes, affecting the quantity and remodeling of ECM proteins. It also has indirect beneficial impacts in terminal chondrocyte maturation through the expression of RUNX-2, the downstream transcription factor associated with the differentiation of chondrocyte to osteoblast development. Different factors including mechanical stress, inflammatory mediators such as TNF-α or IL-1β, aging, fibronectin fragments, and Toll-like membrane receptors can activate NF-kB in OA chondrocytes (Mariani et al., 2014).

The SHED-CM can reduce inflammation in cartilage defects, as it contains anti-inflammatory cytokines (e.g., IL-4 and IL-10), downregulates the NF-kB pathway, and significantly diminishes the expression of proinflammatory cytokines (e.g., IL-1 and TNF-α), besides nitric oxide synthesis (Muhammad et al., 2020; Bar et al., 2021). Additionally, it has been demonstrated that SHED-CM contains a number of growth factors, including TGF-β1, FGF-2, BMP-2 and BMP-4, which are crucial for cartilage regeneration (Bar et al., 2021) TGF-β has the potential to influence SHED activities, including proliferation, collagen turnover, and differentiation. These processes occur *via* TGF-β receptor binding and are differentially controlled by TAK1, MEK/ERK, p38, and ALK5/Smad2/3 signaling (Chang et al., 2020).

Muhammad et al. (2020) in a study on SHED-CM in cartilage regeneration, found that it enhances the expression of aggrecan and type II collagen in OA chondrocytes. The SHED-CM protected cartilage cells by enhancing matrix proteins and suppressing MMP-13 production. This work shows that soluble paracrine components in SHED-CM have an anabolic effect on chondrocytes by downregulating NF-kB signaling and catabolic activity. Ogasawara et al. (2020) studied the effect of SHED-CM on a model of temporomandibular joint osteoarthritis (TMJOA). In this study, it was demonstrated that SHED-CM can hinder cartilage degradation, reduce inflammation, enhance cellular proliferation, promote cartilage regeneration, and prevent TMJOA development through mechanical stress.

### Tooth banking

Accessibility and low invasion concerns are two advantages of deciduous teeth over other tissue or fluid. However, this great resource is only available to youngsters with deciduous teeth. The preservation of these cells is a major challenge when SHED is used therapeutically (Volponi et al., 2010). Since the isolation of SHED is complicated, conservation and banking strategies must be developed for clinical applications. Stem cell cryopreservation has valuable advantages, including long-term storage, differentiation potential retention, contaminants control for safety and quality, and dosage adjustment of cell treatment in therapeutic interventions; therefore, attention has been shifted toward banking (Hubel, 1997; Papaccio et al., 2006). It is important to realize that stem cells may keep their primary properties, such as long-term tissue regeneration after cryopreservation. The use of a magnetic field during freezing has been also shown to boost the vitality of frozen cells. Ice crystals develop during freezing and cause excessive disruptions in osmosis and the plasma membrane (Pilbauerová and Suchánek, 2018). Overall, the application of a magnetic field minimizes cytotoxicity and the dimethyl sulfoxide content in freezing media, resulting in improved cell performance following thawing (Lin et al., 2015). The number of specific stem cell preservation banks for deciduous teeth has increased in North America, the United Kingdom, and India. In 2004, the University of Hiroshima in Japan developed the first dental banks (Kaku et al., 2010). In a study by Zhang et al. (2006) even after freezing, dental pulp stem cells could be employed as a source of multipotent stem cells for tissue regeneration and cell-based treatment. Since the first hematopoietic stem cell transplantation from umbilical cord blood (UCB) bank in 1988, public and family UCB bank have been developed worldwide (Mayani et al., 2020). There are a number of hematologic, pediatric, genetic, immunological, and oncological disorders that have been successfully treated using UBC, as a rich source of hematopoietic stem cells (Lubin and Shearer, 2007). It has been suggested that current banking methods are not valid due to a lack of validated tissue processing regulations (e.g., volume reduction, red blood cell removal, and plasma removal) (Smith and Thomson, 2000). Dental stem cell banking is rapidly finding its place in stem cell-based therapies, similar to UCB which has been cryopreserved for at last 2 decades. The advantages and disadvantages of using umbilical cord stem cells and deciduous teeth are summarized in Table 1.

**TABLE 1:** Advantages and disadvantages of stem cells from umbilical cord blood and SHED.

	Advantages	Disadvantages	References
Umbilical cord blood stem cells banking	1. The collection is simple and painless and not time-consuming	1. Slow engraftment	Moise, 2005; Waller-Wise (2011)
	2. No donor attribution (no risk for mother or child)	2. Large inventory product (high up-front costs; units may become “outdated” due to changes in banking standards	
	3. Higher proliferative capacity	3. Limited cell dose	
	4. Low risk for transmission of infection, Lower rate of acute graft-vs-host disease	4. Autologous donation may have limited benefits owing to hereditary disorders, Storage issues	
	5. Less expensive than bone marrow harvest and less rejection risk		
Stem cells from exfoliated deciduous teeth banking	1. There was no immunological response or tissue rejection, no immunosuppressive treatment was required, and the risk of transmitting disease was considerably decreased	1. Long-term clinical trials are still needed to evaluate the oncogenic potential	Arthur et al. (2008), Mao (2008), Shi et al. (2005), Cordeiro et al. (2008), Mao et al. (2006), Arora et al. (2009)
	2. For both the child and the parent, it is simple and painless	2. The study is mostly focused on animal models, but human research trials are still required to determine the same outcomes in humans	
	3. Less than one-third of the cost of cord blood storage	3. To be applied therapeutically, large numbers are needed	
	4. Not vulnerable to the same ethical considerations as embryonic stem cells	5. Compared to embryonic stem cells, they have a lower potency	
	5. For multiple tissue regeneration such as connective tissues, dental tissues, neural tissue, and bone marrow can be used		

### Challenges

Cell-based therapies have several challenges, including cell implantation time, migration, survival, rejection, and immunological incompatibility (Pearse and Bunge, 2006). On the other hand, the characteristics of self-renewability and plasticity are critical hallmarks of cancer cells. The concept that the transplanted stem cells may lose control and facilitate tumor development is a threatening and irreversible side effect (Knoepfler, 2009; Vicente-Duenas et al., 2009). Although SHED is a valuable autologous resource for restoring damaged tissue, it is only available to children who have lost their deciduous teeth. Therefore, commercial SHED banking, which is relatively new method compared to other types of banking, attempts to keep these important cells and utilizes them to cure disease and restore tissue in adulthood. Meanwhile, dental stem cells are often involved in neoplasia; therefore, mechanisms that allow these stem cells to undergo self-renewal, differentiation, and cancer should be investigated (Xiao and Nasu, 2014). Overall, stem cells provide significant opportunities owing to their differentiation into different lineages and regeneration of multiple tissues. Still, the use of SHED in cartilage tissue engineering has not expanded, and so far, no specific application has been reported (Barry and Murphy, 2004; Barry and Murphy, 2013).

## Conclusion

Cartilage, when severely damaged, cannot restore and regenerate itself due to the absence of nerves, lymph, and arteries (Van Pham, 2017). Because of the unpredictability of conventional surgical procedures and the relative improvement of injured tissue, the use of stem cells and cartilage tissue engineering has received more attention. Currently, stem cell transplantation is a promising approach for cartilage restoration (Orth et al., 2014; Murphy et al., 2020). The MSCs are the most reliable source in stem cell-based regenerative medicine. As reviewed in this article, SHED exhibits phenotypes of MSCs, such as expression of MSCs-related markers, self-renewal, multipotency, and immunoregulatory effects. This cellular source can be recruited for cell-based regeneration in cartilage disorders.
